# Death on New Year’s Eve caused by illegal fireworks—a firework shell

**DOI:** 10.1007/s12024-023-00633-2

**Published:** 2023-05-12

**Authors:** Julia Ulbricht, Elke Doberentz, Burkhard Madea

**Affiliations:** Institute of Legal Medicine, University Hospital Bonn, Stiftsplatz 12, 53111 Bonn, Germany

**Keywords:** Firework shell, Ball shell, New Year’s Eve, Bullet, Skin lacerations, Explosive forces

## Abstract

During the turn of the year, injuries caused by fireworks occur in Germany every year. According to the professional associations, the Unfallkrankenhaus Berlin, for example, treats an average of 50 injuries caused by fireworks on New Year’s Eve. Patients come with burns, soft tissue injuries, or fractures; eyes and hands are particularly frequently affected. Again and again, there are also very serious or even fatal injuries. The background is usually the improper or illegal use of larger fireworks. Smaller fireworks such as sparklers, bangers, or smaller rockets are available in Germany in most supermarkets, and their use is permitted from the age of 12 or 18. However, the use of larger fireworks in Germany requires proof of an official permit to handle pyrotechnic objects, which is why they are often acquired abroad. The following report describes such a case. Shortly after the turn of the year 2022, a young man died as a result of an explosive effect on the facial skull after using an illegal firework shell. The case is discussed with regard to the autopsy findings, the possible cause of the accident, and the type of firework used.

## Introduction

In almost all countries, fireworks are used for festivities on a wide variety of cultural occasions. Unfortunately, fireworks as recreational explosives are responsible for many accidents around the world. According to the professional associations, the Unfallkrankenhaus Berlin, for example, treats an average of 50 injuries caused by fireworks on New Year’s Eve. Patients come with burns, soft tissue injuries, or fractures; eyes and hands are particularly frequently affected [1]. Again and again, there are also very serious or even fatal injuries. The background is usually the improper or illegal use of larger fireworks. Smaller fireworks such as sparklers, bangers, or smaller rockets are available in Germany in most supermarkets, and their use is permitted from the age of 12 or 18. However, the use of larger fireworks in Germany requires proof of an official permit to handle pyrotechnic objects, which is why they are often acquired illegally [2].

According to the US Consumer Product Safety Commission (CPSC) in 2020, at least 18 people died in firework-related incidents and about 15,600 people were treated in hospital emergency departments for firework injuries in the USA. Of the 18 deaths, 8 of the victims had consumed alcohol or drugs prior to the incident [3]. In Northern Ireland, 15 persons were admitted to emergency rooms with firework-related injuries in 2015, and in Denmark, 4447 firework-related injuries were recorded between 1995 and 2007, of which five died [4].

And even apart from accidents caused by legal fireworks or self-elaborated and homemade bombs, fireworks have been used as a method of suicide, e.g., by intraoral detonation of a firecracker, and there has even been a reported case of homicide where the victim was killed by a firework modified with stone, nails, and glass [5–7].

## Case description

Shortly after midnight, on the first of January 2022, an explosion described by witnesses occurred in a rural area. A 37-year-old and a 39-year-old man had left a larger group only a few minutes earlier when there was a sudden loud explosion. The 39-year-old man was injured. The younger man was resuscitated, but he died from his severe injuries at the scene of the accident.

**Fig. 1:**
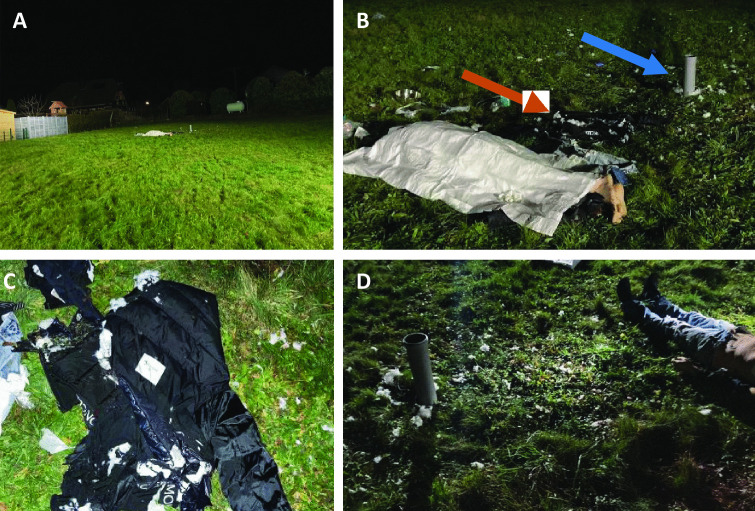
**A-D** Death scence in a large garden, a gray rain pipe stuck in the ground (blue arrow) and filling material of a jacket (orange arrow) where found next to the body

The man died in a large garden area near his house (Fig. 1a). About 2 m next to the young man’s body, a gray rain pipe was stuck in the ground of the meadow, which had apparently been placed there intentionally. Next to it was a jacked with burnt fabric as well as white flakes of the filling material of the jacket (Fig. 1b–d).

**Fig. 2:**
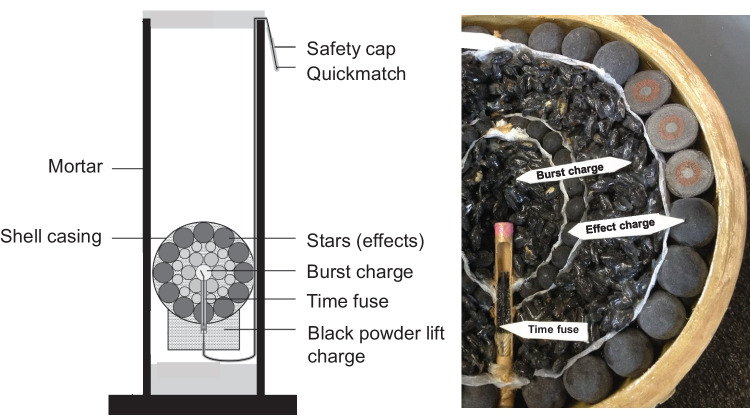
Construction and cross section of a ball shell

According to the police, no remains of an explosive device could be found. Prior to the autopsy, the police assumed that it was most likely rocket batteries or a firework shell. Even though no fragments of an explosive device were found by the police, the setup of the fireworks’ launch site with a mortar-like launch tube as well as the results of the autopsy (see below) strongly suggest the use of a firework shell device. Firework shells belong to the category of aerial fireworks, which may be shaped as a spherical shell. The container is usually made of cardboard and a string with which the grenade is lowered into a tube. They are usually launched into the sky with the help of a black powder charge from tubes (so-called mortars). When fired, a time fuse located on the underside of the bullet casing is activated, so that the casing is ideally ignited at the so-called culmination point. In the process, the disintegrator charge ruptures the outer container, usually made from pasted paper, driving the charge apart in a spherically symmetrical manner and igniting the effects (Fig. 2) [8].

## Autopsy findings

Three main types of injury could be differentiated during postmortem examination.

- Blast injury
- Projectile injury
- Burn injury

### Blast injury

**Fig. 3:**
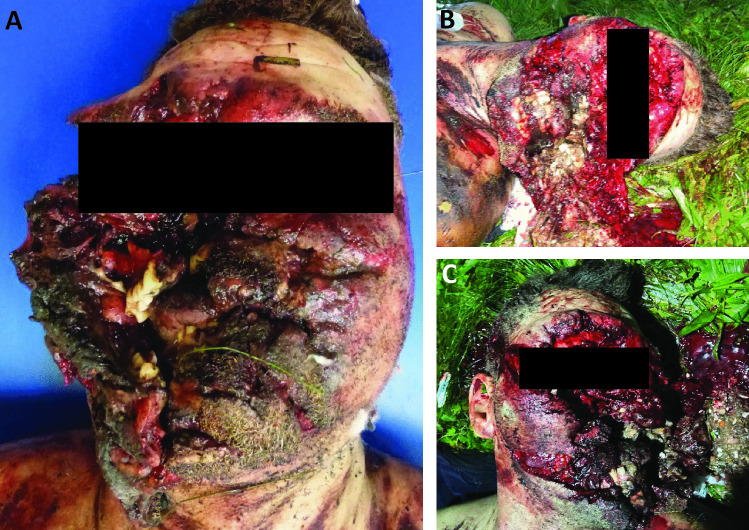
Massive destruction of the soft tissue of the face, during autopsy (**A**) and at death scene (**B**, **C**)

**Fig. 4:**
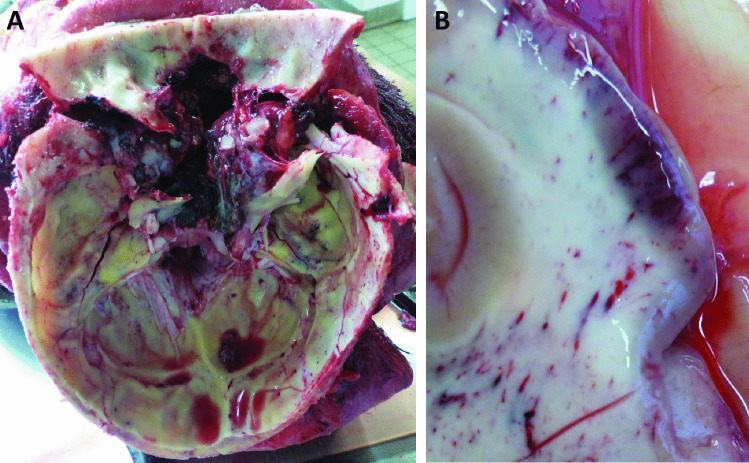
Shattered fracture of the anterior cranial fossa with transition to the middle cranial fossa (**A**). Contusion hemorrhage on the right temporal lobe (**B**)

External examination revealed massive destruction of almost all facial tissue; the skin was lacerated. Eyes, nose, and oral cavity were mutilated and no longer depictable (Fig. 3a–c). The wound edges were frayed and partially blackened with soot. The facial skull was shattered in fragments. On internal inspection, the entire anterior cranial fossa with transition to the middle cranial fossa as well as the right temporal and parietal bones showed comminuted fractures (Fig. 4a). The right temporal and left parietal lobes had contusion hemorrhages (Fig. 4b), and there was a thin film of blood under the soft meninges. The left and right lungs were hyperinflated.

### Projectile injury

**Fig. 5:**
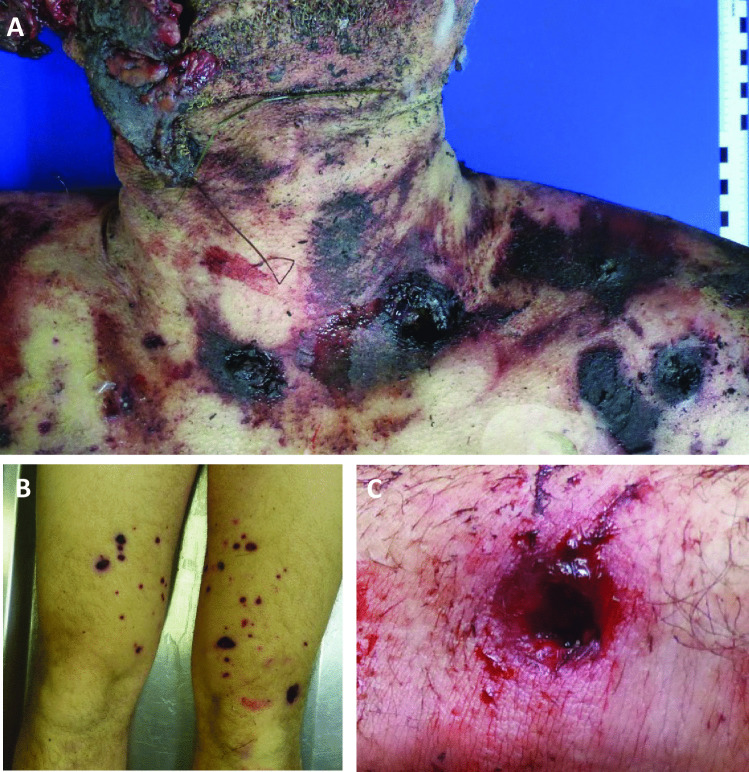
Roundish skin lacerations on the chest (**A**) and both tighs (**B**) shaped like gunshot wounds (**C**)

**Fig. 6:**
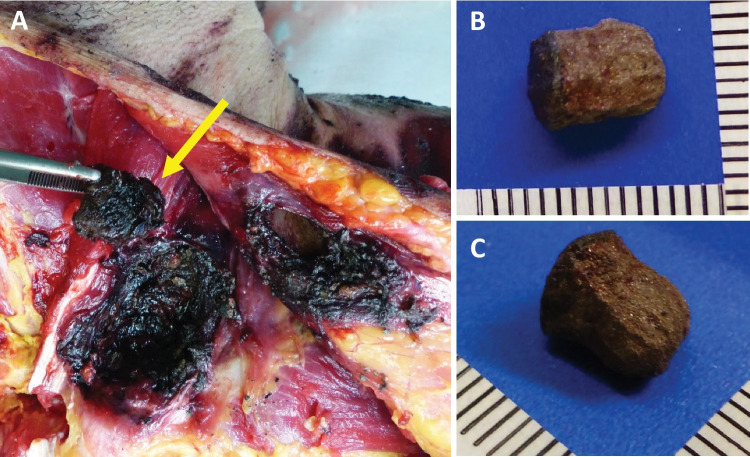
Skin laceration blackened with soot where a piece of cardboard (yellow arrow) (**A**) and two metal like particles were found (**B**, **C**)

On both anterior sides of the chest near the shoulder level, on the outer side of the left upper arm on both thighs anteriorly or internally, and on the outer side of the forearms, there were several roundish skin lacerations looking like gunshot wounds (Fig. 5). On the left side of the neck, there was also a roundish skin transection in which cardboard and two metal-like particles were found (Fig. 6).

### Burn injuries

A burn with detachment of the epidermis and soot-like discoloration of the skin were also noted on the outer upper arm.

The lividity appeared very sparse; beard and head hairs were scorched. The tongue was blackened with soot, and there was gastric content in the airways of both lungs and trachea. The brain weight was 1320 g.

**Fig. 7:**
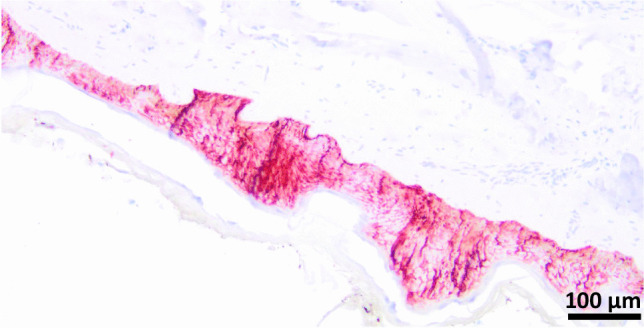
Expression of aquaporin 3 in the skin laceration from the chin

The determination of blood alcohol concentration showed a value of 1.18‰. Immunohistochemical examination of the round skin lesions (face, upper arm, chest) showed a positive expression of the vitality marker aquaporin 3 (Fig. 7) [9, 10].

## Discussion

The extent of injuries from explosive forces decreases exponentially with distance from detonation. Injuries at close range result in characteristic injuries with amputation, body dissection, and massive tissue defects [11]. In the present case, there were severe soft tissue injuries as a sign of direct injury to the body by material of the explosive device, so it can be assumed that the explosive device was close by and detonated in the area of the head. The additional injuries inside the cranial cavity and the skull (fractures, contusion hemorrhages) speak for the massive impact of violence. In the case of blast injuries, different types of injuries can be distinguished depending on the point of origin. In addition to primary injuries caused by the detonation wave at the interface of air and tissue, there may be secondary projectile injuries caused by fragments added to the explosive device or by projectiles from the surroundings. The ballistic effect of the projectiles depends on the mass, velocity, and shape of the fragments and can therefore range from very small penetrations to large lacerations. Often the fragments are smaller and irregular in shape, so there is a strong deceleration into air and tissue despite high initial velocity. The penetration depth and the extent of injury of the projectile wounds are therefore often limited. In the present case, the projectile injuries were also caused by fragments from the ball bomb [12].

The roundish skin perforations were morphologically similar to gunshot wounds with a central non-adaptable substance defect and an epidermis-free marginal zone (abrasion ring). Most of the skin lacerations had a black rim. Considering the effective charge of projectile bombs, which generally consists of metal salts and mainly black powder, it is not surprising that the skin changes can hardly be distinguished from an injury caused by a usual projectile. The injury pattern would be comparable to a bullet penetration caused by a close range shot, whereby the projectiles usually get stuck on the opposite side of the body beneath the skin or harder resistances like bones. In the present case, the fragment was small and more irregularly shaped than usual projectiles; the dispersal of the effect charge therefore only led to a small penetration depth of the tissue. Besides the effect charge, a piece of cardboard discolored with soot was found in the skin laceration. This was most likely the outer shell of the bullet bomb, which is usually made of cardboard. Hot explosive gases and resulting secondary fires can also cause thermal injuries such as the burn to the young man’s upper arm [13, 14].

**Fig. 8:**
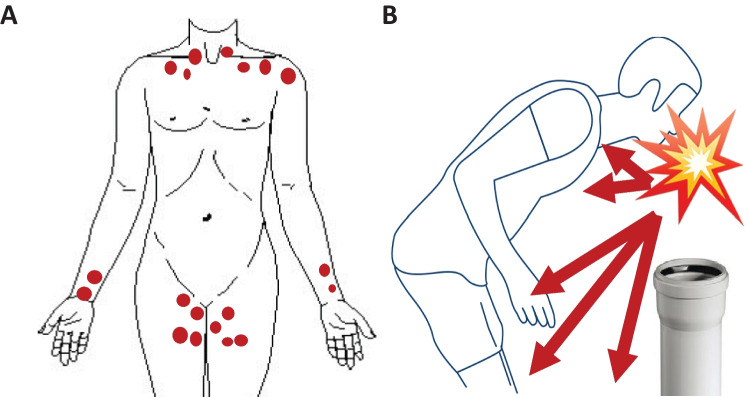
Schematic illustration of the injury pattern of the round skin lacerations (**A**). Possible body posture at the time of the explosion (**B**)

Why the accident occurred could not be clarified by the police investigation. From the investigation documents, it could only be inferred that the fireworks, which had been used that evening, had previously been purchased through a “seller via WhatsApp” due to the sales ban. On the basis of the injury pattern of the roundish skin defects (Fig. 8a), the impression of a bent posture arises, in which the hands may have been supported on the thighs, which could have brought the respective localizations “closer together” and protected the abdomen so to speak (Fig. 8b).

Such a posture could have been adopted, for example, when igniting the explosive device, while the ball bomb ignited too quickly and immediately, for example. Also, which is more likely, a renewed “look down the tube” because the firework did not ignite immediately would be conceivable. The detected blood alcohol concentration would support this tragic behavior.

## Key points

1. The death of a young man on New Year’s Eve caused by use of illegal fireworks.
2. Autopsy findings could reveal the type of firework used.
3. The mechanism of a firework shell is explained.
4. External examination revealed several roundish skin lesions looking like gunshot wounds.
5. Injuries caused by blast effect and the similarities with gunshot wounds are discussed as well as the posture the man may have adopted.
